# Crystal structure of 4-carbamoylpyridinium chloride

**DOI:** 10.1107/S2056989016003340

**Published:** 2016-03-04

**Authors:** Simon M. Fellows, Timothy J. Prior

**Affiliations:** a Department of Chemistry, University of Hull, Cottingham Road, Hull HU6 7RX, United Kingdom

**Keywords:** crystal structure, isonicotinamide, hydro­chloride, hydrogen bonding

## Abstract

The hydro­chloride salt of isonicotinamide, C_6_H_7_N_2_O^+^·Cl^−^, has been synthesized from a dilute solution of hydro­chloric acid in aceto­nitrile. The compound displays monoclinic symmetry (space group *C*2/*c*) at 150 K, similar to the related hydro­chloride salt of nicotinamide. The asymmetric unit contains one protonated isonicotinamide mol­ecule and a chloride anion. An array of hydrogen-bonding inter­actions, including a peculiar bifurcated pyridinium–chloride inter­action, results in linear chains running almost perpendicularly in the [150] and [1-50] directions within the structure. A description of the hydrogen-bonding network and comparison with similar compounds are presented.

## Abstract 2

The hydro­chloride salt of isonicotinamide has been synthesized from a dilute solution of hydro­chloric acid in aceto­nitrile and displays monoclinic symmetry (space group *C*2/*c*) at 150 K, similar to the related hydro­chloride salt of nicotinamide. An array of hydrogen-bonding inter­actions, including a peculiar bifurcated pyridinium–chloride inter­action, results in linear chains.

## Chemical context   

Often compounds which exhibit a desirable biological function may not possess the correct physical properties for practical usage. The ability to manipulate the properties of a compound in a controlled manner, while maintaining the biological activity, is one of the ultimate goals of crystal engineering (Desiraju *et al.*, 2011 ▸). Converting a biologically active compound into its hydro­chloride salt has proven a successful technique in this regard (Byrn *et al.*, 1999 ▸). The structural determination and analysis of neutral, co-crystalline, and salt forms of various mol­ecules is necessary in order to expand the library of reliable tools that can be used in the design of new materials.
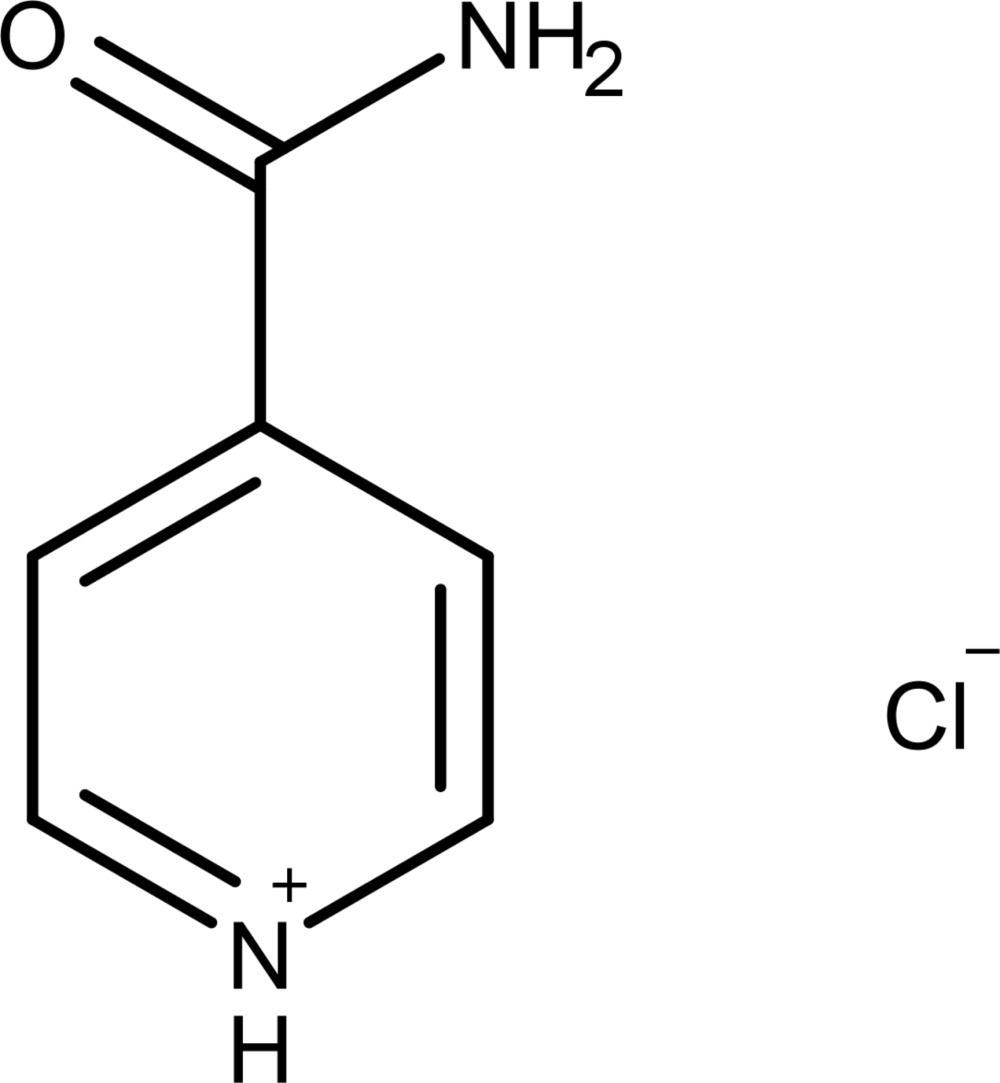


Isonicotinamide is a useful compound in crystal engineering as it possesses amide and pyridyl groups which have the capability to form a plethora of well established and predictable hydrogen-bonding arrangements (Bhogala *et al.*, 2004 ▸). It also displays polymorphism in the solid state on account of its flexible hydrogen-bonding capacity (Aakeröy *et al.*, 2003 ▸; Li *et al.*, 2011 ▸). As a result, the mol­ecule has been heavily investigated by many groups as a co-crystal former in lots of different scenarios (Vishweshwar *et al.*, 2003 ▸; Bhogala *et al.*, 2005 ▸; Aakeröy *et al.*, 2007 ▸; Thompson *et al.*, 2011 ▸; Tothadi & Desiraju, 2012 ▸; Dubey & Desiraju, 2014 ▸; Kerr *et al.*, 2015 ▸). The compound is also of mild pharmaceutical inter­est given its similarity to nicotinamide, the amide of Vitamin B_3._ The hydro­chloride salt of this mol­ecule has been synthesized and the structure determined, the results of which are discussed herein.

## Structural commentary   

**Figure 1:**
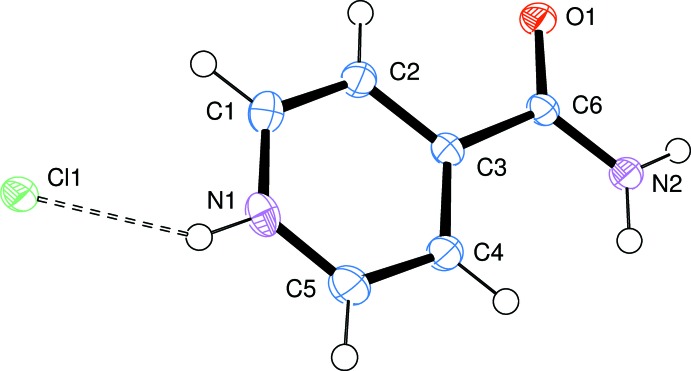
The asymmetric unit of the title compound, showing the atom-numbering scheme. Displacement ellipsoids are drawn at the 50% probability level. The dashed line represents a hydrogen bond.

The asymmetric unit of the title compound, shown in Fig. 1 ▸, consists of one chloride anion and a protonated isonicotinamide cation, confirmed by the identification of a proton 0.9 Å from the pyridine N atom in a difference Fourier map. The isonicotinamide cation is planar: the root-mean-square deviation of the pyridinium ring is 0.0062 Å, with an angle of 1.3 (2)° between the planes of the pyridinium and amide moieties. Analysis of structures in the Cambridge Structural Database (CSD, Version 5.37, update November 2015; Groom & Allen, 2014 ▸) containing the 4-carbamoylpyridinium cation show that the angle between the amide and pyridinium planes can take any value between 0 and 50° with no distinct configurational preference.

## Supra­molecular features   

**Figure 2:**
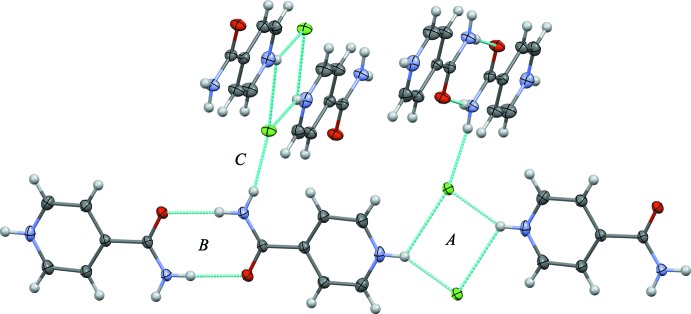
A view of the hydrogen bonding arrangements within 4-carbamoylpyridinium chloride, showing the pyridinium–chloride (*A*) amide–amide (*B*) and amine–chloride (*C*) inter­actions. Hydrogen bonds are drawn as light-blue dashed lines. Possible C—H⋯*X* inter­actions have been omitted.

The most inter­esting feature of the crystal structure is the inter­molecular inter­action between the pyridinium groups and chloride ions. Two pyridinium protons form bifurcated hydrogen bonds to two chloride ions, forming an 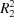
(4) ring motif which is positioned on a centre of inversion, as shown in Fig. 2 ▸ (*A*). The inter­action is similar to those found in many tetra­halometallate [*MX*_4_]^2−^ compounds, though primarily when the [*MX*_4_]^2−^ unit is planar (Adams *et al.*, 2006 ▸). It is also encountered in some hydro­chloride salts of mol­ecules incorporating a pyridinium group (Nattinen & Rissanen, 2003 ▸; Zhao *et al.*, 2008 ▸). The two unique N—H⋯Cl hydrogen bonds formed by each proton in this arrangement are usually of similar length, though one is distinctly longer than the other in this compound [N⋯Cl = 3.0416 (12) Å and 3.4882 (13) Å]. The CSD (Version 5.37, update November 2015; Groom & Allen, 2014 ▸) reveals 426 structures of the hydro­chloride salts of mol­ecules incorporating a pyridinium group. 21 of these display bifurcated pyridinium–chloride hydrogen bonds, of which only 16 possess the 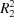
(4) ring motif. None of these simultaneously show the same asymmetry in the hydrogen-bond lengths (ratio of two N⋯Cl lengths = 1.147; ratio of H⋯Cl lengths = 1.317), and planarity (r.m.s.d. = 0.0151 Å) of the inter­action as the title compound. Given the lack of examples of bifurcated hydrogen bonds in this type of material, it seems likely that it is a result of maximizing the other possible inter­molecular inter­actions for a given system, where the bifurcation is a compromise. In other words, one short, linear N—H⋯Cl bond is ideal, though bifurcation is energetically more favourable than a single, weaker inter­action.

**Figure 3:**
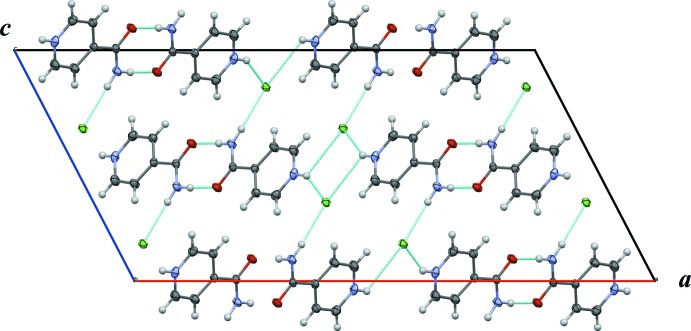
Crystal packing diagram of 4-carbamoylpyridinium chloride viewed along the *b* axis. Hydrogen bonds are drawn as light-blue dashed lines.

The amine group of the amide moiety is directed toward the carboxyl group of an adjacent mol­ecule related by a centre of inversion; this shows the existence of a classic 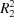
(8) amide-amide inter­action, seen in Fig. 2 ▸ (*B*). The combination of the two ring inter­actions form 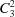
(16) chains running in the [150] and [1
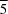
0] directions, and are related by a 2_1_ screw axis. The two chain directions are almost perpendicular to each other (82°) and are held in this respective orientation by a 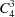
(10) inter­action which incorporates the 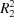
(8) and 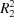
(4) motifs, and hydrogen bonds between the protons not involved in the amide–amide inter­actions and the chloride ions of neighbouring chains, as shown in Fig. 2 ▸ (*C*). For the overall packing arrangement, see Fig. 3 ▸. There is some evidence of C—H⋯*X* hydrogen bonds (Table 1 ▸); however, these only seem to reinforce the stronger inter­actions discussed above and their role in determining the crystal packing in this compound is unclear.

The related salt of nicotinamide contains the same amide–amide inter­actions as in the title compound, though neither 3- or 4-carb­oxy­pyridinium chloride show the equivalent di­carb­oxy­lic acid inter­action. (Gubin *et al.*, 1989 ▸; Slouf, 2001 ▸; Adams *et al.*, 2006 ▸). The chloride ions in these structures act only as hydrogen-bond acceptor atoms between donor atoms of mol­ecules in the same chain, with no further inter­actions between the chains. It would be inter­esting to see how these almost classic co-crystal formers would behave when crystallized with the hydro­chloride salts of other mol­ecules, and whether the same hydrogen-bonding arrangements persist.

## Related structures   

For the crystal structure of 4-carbamoylpyridinium di­hydro­gen phosphate, see Gholivand *et al.* (2007 ▸); for 4-carbamoyl­pyridinium perchlorate, see Chen (2009 ▸); for nicotinamide hydro­chloride, see Gubin *et al.* (1989 ▸); for nicotinic acid hydro­chloride, see Slouf (2001 ▸); and for isonicotinic acid hydro­chloride and a comprehensive study on the tetra­chloro­platinate and palladate salts of similar pyridinium compounds, see Adams *et al.* (2006 ▸).

## Synthesis and crystallization   

Hydro­chloric acid (0.08 ml, 12 *M*) in aceto­nitrile (3 ml) was added to isonicotinamide (0.244 g, 2 mmol) dissolved in aceto­nitrile (25 ml). The resultant white mixture was heated until the precipitate dissolved and the solution left to evaporate slowly over several days, resulting in the formation of large colourless block-shaped crystals of the title compound.

## Refinement   

Crystal data, data collection and structure refinement details are summarized in Table 2 ▸. Hydrogen atoms were readily identified in difference Fourier maps. The pyridinium hydrogen atom was positioned in a geometrically optimized position; N1—H1*A* was constrained to a value of 0.88 Å with an su of 0.03 Å. C-bound H atoms were sited with a riding model and the C—H distance refined subject to the restraint that all C—H distances should be the same with an su of 0.03 Å. The amide hydrogen atoms were refined freely subject to restraint that the two N—H bond lengths were equal with an su of 0.03 Å and the H⋯H distance was 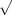
3 × l_N–H_ (su 0.03 Å) to set the H—N—H angle to 120°.

## Supplementary Material

Crystal structure: contains datablock(s) I. DOI: 10.1107/S2056989016003340/zl2658sup1.cif

Structure factors: contains datablock(s) I. DOI: 10.1107/S2056989016003340/zl2658Isup2.hkl

Supporting information file. DOI: 10.1107/S2056989016003340/zl2658Isup3.mol

Web address for enhanced figure. DOI: 10.1107/S2056989016003340/zl2658sup4.txt

Supporting information file. DOI: 10.1107/S2056989016003340/zl2658Isup5.cml

CCDC reference: 1455942

Additional supporting information:  crystallographic information; 3D view; checkCIF report

## Figures and Tables

**Table 1:** Hydrogen-bond geometry (Å, °)

*D*—H⋯*A*	*D*—H	H⋯*A*	*D*⋯*A*	*D*—H⋯*A*
C1—H1⋯O1^i^	0.95	2.74	3.295 (2)	118
C2—H2⋯O1^i^	0.92	2.61	3.2205 (18)	124
C4—H4⋯Cl1^ii^	0.95	2.59	3.5377 (15)	172
C5—H5⋯Cl1^iii^	0.93	2.62	3.3284 (15)	133
N1—H1*A*⋯Cl1	0.89	2.24	3.0416 (12)	149
N1—H1*A*⋯Cl1^iii^	0.89	2.95	3.4882 (13)	121
N2—H2*A*⋯O1^iv^	0.88 (2)	2.02 (2)	2.8892 (15)	174 (2)
N2—H2*B*⋯Cl1^ii^	0.87 (2)	2.33 (2)	3.1907 (13)	169 (2)

Symmetry codes: (i) 
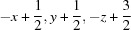
; (ii) 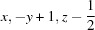; (iii) 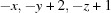; (iv) 
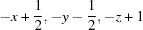
.

**Table 2:** Experimental details

Crystal data	
Chemical formula	C_6_H_7_N_2_O^+^·Cl^−^
*M*_r_	158.59
Crystal system, space group	Monoclinic, *C*2/*c*
Temperature (K)	150
*a*, *b*, *c* (Å)	24.960 (2), 5.1055 (4), 12.4664 (9)
β (°)	117.545 (5)
*V* (Å^3^)	1408.6 (2)
*Z*	8
Radiation type	Mo *K*α
μ (mm^−1^)	0.47
Crystal size (mm)	0.34 × 0.28 × 0.26

Data collection	
Diffractometer	Stoe *IPDS* 2
Absorption correction	Analytical (*X-RED* and *X-SHAPE*; Stoe & Cie, 2012 ▸)
*T*_min_, *T*_max_	0.861, 0.913
No. of measured, independent and observed [*I* > 2σ(*I*)] reflections	4768, 1865, 1520
*R*_int_	0.047
(sin θ/λ)_max_ (Å^−1^)	0.685

Refinement	
*R*[*F* ^2^ > 2σ(*F* ^2^)], *wR*(*F* ^2^), *S*	0.034, 0.093, 1.01
No. of reflections	1865
No. of parameters	104
No. of restraints	8
H-atom treatment	H atoms treated by a mixture of independent and constrained refinement
Δρ_max_, Δρ_min_ (e Å^−3^)	0.45, −0.48

Computer programs: *X-AREA* (Stoe & Cie, 2012 ▸), *X-RED* (Stoe & Cie, 2012 ▸), *SHELXT* (Sheldrick, 2015*a* ▸), *SHELXL2014* (Sheldrick, 2015*b* ▸), *SHELXTL* (Sheldrick, 2008 ▸) and *Mercury* (Macrae *et al.*, 2008 ▸).

## supplementary crystallographic information

### Crystal data

**Table:**

C_6_H_7_N_2_O^+^·Cl^−^	*F*(000) = 656
*M**_r_* = 158.59	*D*_x_ = 1.496 Mg m^−^^3^
Monoclinic, *C*2/*c*	Mo *K*α radiation, λ = 0.71073 Å
*a* = 24.960 (2) Å	Cell parameters from 5016 reflections
*b* = 5.1055 (4) Å	θ = 3.7–59.1°
*c* = 12.4664 (9) Å	µ = 0.47 mm^−^^1^
β = 117.545 (5)°	*T* = 150 K
*V* = 1408.6 (2) Å^3^	Block, colourless
*Z* = 8	0.34 × 0.28 × 0.26 mm

### Data collection

**Table:**

Stoe IPDS 2 diffractometer	1865 independent reflections
Radiation source: fine-focus sealed X-ray tube	1520 reflections with *I* > 2σ(*I*)
Detector resolution: 6.67 pixels mm^-1^	*R*_int_ = 0.047
ω scans	θ_max_ = 29.2°, θ_min_ = 1.8°
Absorption correction: analytical (*X-RED* and *X-SHAPE*; Stoe & Cie, 2012)	*h* = −34→34
*T*_min_ = 0.861, *T*_max_ = 0.913	*k* = −6→6
4768 measured reflections	*l* = −17→17

### Refinement

**Table:**

Refinement on *F*^2^	8 restraints
Least-squares matrix: full	Hydrogen site location: mixed
*R*[*F*^2^ > 2σ(*F*^2^)] = 0.034	H atoms treated by a mixture of independent and constrained refinement
*wR*(*F*^2^) = 0.093	*w* = 1/[σ^2^(*F*_o_^2^) + (0.0636*P*)^2^] where *P* = (*F*_o_^2^ + 2*F*_c_^2^)/3
*S* = 1.01	(Δ/σ)_max_ = 0.001
1865 reflections	Δρ_max_ = 0.45 e Å^−^^3^
104 parameters	Δρ_min_ = −0.48 e Å^−^^3^

### Special details

**Table:**

Geometry. All e.s.d.'s (except the e.s.d. in the dihedral angle between two l.s. planes) are estimated using the full covariance matrix. The cell e.s.d.'s are taken into account individually in the estimation of e.s.d.'s in distances, angles and torsion angles; correlations between e.s.d.'s in cell parameters are only used when they are defined by crystal symmetry. An approximate (isotropic) treatment of cell e.s.d.'s is used for estimating e.s.d.'s involving l.s. planes.
Refinement. Refinement of **F**^2^ against ALL reflections. The weighted **R**-factor *wR* and goodness of fit **S** are based on **F**^2^, conventional **R**-factors **R** are based on **F**, with **F** set to zero for negative **F**^2^. The threshold expression of **F**^2^ > σ(**F**^2^) is used only for calculating **R**-factors(gt) *etc*. and is not relevant to the choice of reflections for refinement. **R**-factors based on **F**^2^ are statistically about twice as large as those based on **F**, and **R**-factors based on ALL data will be even larger.

### Fractional atomic coordinates and isotropic or equivalent isotropic displacement parameters (Å^2^)

**Table:**

	*x*	*y*	*z*	*U*_iso_*/*U*_eq_	
Cl1	0.05337 (2)	1.08640 (7)	0.66004 (3)	0.02094 (12)	
C1	0.14210 (7)	0.6050 (3)	0.62858 (14)	0.0235 (3)	
H1	0.1565 (2)	0.7082 (17)	0.7004 (12)	0.028*	
C2	0.17821 (6)	0.4124 (3)	0.61830 (13)	0.0206 (3)	
H2	0.2164 (7)	0.3837 (6)	0.6800 (11)	0.025*	
C3	0.15607 (5)	0.2616 (3)	0.51318 (11)	0.0175 (3)	
C4	0.09760 (6)	0.3065 (3)	0.42116 (13)	0.0236 (3)	
H4	0.0814 (3)	0.2027 (17)	0.3497 (12)	0.028*	
C5	0.06356 (7)	0.5048 (3)	0.43560 (14)	0.0270 (3)	
H5	0.0247 (7)	0.5390 (7)	0.3747 (12)	0.032*	
C6	0.19697 (6)	0.0497 (3)	0.50687 (12)	0.0173 (3)	
N1	0.08665 (6)	0.6468 (2)	0.53714 (12)	0.0228 (3)	
H1A	0.0642 (4)	0.775 (2)	0.54449 (16)	0.027*	
N2	0.17626 (5)	−0.0961 (2)	0.40762 (11)	0.0209 (3)	
O1	0.24768 (4)	0.0199 (2)	0.59516 (9)	0.0228 (2)	
H2A	0.2004 (8)	−0.216 (4)	0.4039 (16)	0.027*	
H2B	0.1426 (8)	−0.069 (4)	0.3420 (17)	0.027*	

### Atomic displacement parameters (Å^2^)

**Table:**

	*U*^11^	*U*^22^	*U*^33^	*U*^12^	*U*^13^	*U*^23^
Cl1	0.01780 (17)	0.0231 (2)	0.01907 (18)	0.00607 (12)	0.00610 (13)	−0.00009 (13)
C1	0.0261 (7)	0.0220 (7)	0.0249 (7)	−0.0003 (5)	0.0138 (6)	−0.0034 (6)
C2	0.0179 (6)	0.0215 (7)	0.0208 (6)	0.0012 (5)	0.0075 (5)	−0.0015 (5)
C3	0.0171 (6)	0.0167 (6)	0.0190 (6)	0.0021 (5)	0.0087 (5)	0.0014 (5)
C4	0.0195 (6)	0.0255 (8)	0.0212 (6)	0.0065 (5)	0.0056 (5)	−0.0034 (6)
C5	0.0207 (7)	0.0294 (8)	0.0277 (7)	0.0091 (6)	0.0086 (6)	0.0003 (6)
C6	0.0155 (6)	0.0170 (7)	0.0190 (6)	0.0022 (5)	0.0075 (5)	0.0013 (5)
N1	0.0236 (6)	0.0195 (6)	0.0303 (7)	0.0055 (5)	0.0166 (5)	0.0003 (5)
N2	0.0173 (5)	0.0217 (6)	0.0198 (6)	0.0061 (4)	0.0052 (5)	−0.0024 (5)
O1	0.0170 (4)	0.0250 (6)	0.0202 (5)	0.0067 (4)	0.0034 (4)	−0.0015 (4)

### Geometric parameters (Å, º)

**Table:**

C1—N1	1.342 (2)	C4—H4	0.951 (16)
C1—C2	1.3795 (19)	C5—N1	1.336 (2)
C1—H1	0.955 (16)	C5—H5	0.931 (18)
C2—C3	1.3948 (19)	C6—O1	1.2443 (17)
C2—H2	0.918 (16)	C6—N2	1.3271 (18)
C3—C4	1.3964 (18)	N1—H1A	0.894 (14)
C3—C6	1.5141 (18)	N2—H2A	0.876 (16)
C4—C5	1.3858 (19)	N2—H2B	0.871 (18)
			
N1—C1—C2	119.73 (14)	N1—C5—C4	119.84 (14)
N1—C1—H1	120.1	N1—C5—H5	120.1
C2—C1—H1	120.1	C4—C5—H5	120.1
C1—C2—C3	119.26 (13)	O1—C6—N2	123.66 (12)
C1—C2—H2	120.4	O1—C6—C3	118.43 (12)
C3—C2—H2	120.4	N2—C6—C3	117.91 (12)
C2—C3—C4	119.39 (12)	C5—N1—C1	122.81 (12)
C2—C3—C6	117.34 (11)	C5—N1—H1A	118.6
C4—C3—C6	123.25 (12)	C1—N1—H1A	118.6
C5—C4—C3	118.94 (14)	C6—N2—H2A	117.5 (12)
C5—C4—H4	120.5	C6—N2—H2B	125.3 (12)
C3—C4—H4	120.5	H2A—N2—H2B	116.7 (16)

### Hydrogen-bond geometry (Å, º)

**Table:**

*D*—H···*A*	*D*—H	H···*A*	*D*···*A*	*D*—H···*A*
C1—H1···O1^i^	0.95	2.74	3.295 (2)	118
C2—H2···O1^i^	0.92	2.61	3.2205 (18)	124
C4—H4···Cl1^ii^	0.95	2.59	3.5377 (15)	172
C5—H5···Cl1^iii^	0.93	2.62	3.3284 (15)	133
N1—H1*A*···Cl1	0.89	2.24	3.0416 (12)	149
N1—H1*A*···Cl1^iii^	0.89	2.95	3.4882 (13)	121
N2—H2*A*···O1^iv^	0.88 (2)	2.02 (2)	2.8892 (15)	174 (2)
N2—H2*B*···Cl1^ii^	0.87 (2)	2.33 (2)	3.1907 (13)	169 (2)

Symmetry codes: (i) −*x*+1/2, *y*+1/2, −*z*+3/2; (ii) *x*, −*y*+1, *z*−1/2; (iii) −*x*, −*y*+2, −*z*+1; (iv) −*x*+1/2, −*y*−1/2, −*z*+1.
